# Supplementary material for: Phytase and phosphate-solubilizing Bacteria addition improves P availability and uptake by maize in low-phosphorus red soil
Source: PLoS One. 2026 May 4;21(5):e0347410. doi: 10.1371/journal.pone.0347410 (PMC13138749; doi:10.1371/journal.pone.0347410)
Supplement: Attachment — Submitted filename: Comments.docx [file pone.0347410.s001.docx]

Comments

The paper addresses an important topic related to phytase and phosphate-solubilizing bacteria for mediating P activation in maize rhizosphere soil and improving P uptake by maize in low-phosphorus red soil. The manuscript is well structured and rich in information. However, several minor revisions are needed, and one of the most important issues concerns the replicability of the results, since the number of repetitions is low. Overall, the document merits publication after these minor revisions.

- Please add line numbers to facilitate comment referencing.

Abstract

- Please italicize *Bacillus* in “phosphate-solubilizing *Bacillus*”.

Introduction

- In the sentence “Representative genera such as bacillus, pseudomonas, and rhizobium enhance P availability”, please capitalize the first litter of genus names and italicize them: *Bacillus*, *Pseudomonas*, and *Rhizobium*.
- Last paragraph: “phosphate-solubilizing bacteria (*Bacillus velezensis*) (PSB)” *Bacillus velezensis* is a species name and must always be italicized.

Materials and methods

- “The maize (Zea mays L.) cultivar…” *Zea mays* must be italicized.
- “a single colony of B. velezensis from a -80 °C stock” *B. velezensis* must be italicized.
- “to prepare a high-density seed culture.” This expression is unclear. Do you mean a high-concentration bacterial suspension? Please clarify.
- “inoculated with or without Bacillus velezensis” Use *B. velezensis*, italicized.
- Replication issue: “6 treatments × 4 replicates = 24 pots” is statistically low, especially for same plante like *Zea mays*. Even if 3–5 seeds were sown per pot, the replication number remains low and may affect experimental reproducibility. Please address or justify this.
- “Bacterial inoculation was performed by applying 50 mL of B. velezensis suspension (≈10¹⁰ CFU·mL⁻¹)”: *B. velezensis* must be italicized. And the concentration 10¹⁰ CFU·mL⁻¹ is very high; most studies use 10⁸ CFU·mL⁻¹ (or 10⁸ CFU·g⁻¹ of soil). Please justify your choice.
- Mycorrhizal colonization: Please explain how colonization was quantified (clearing, staining method, microscopic scoring method, etc.).

Results

- Table 1: Add a table title.
- Table 1 and Table 2: Add the explanation under the tables: “-Po indicates no organic P applied; Po indicates organic P applied. CK = control; PHY = phytase addition; PSB = phosphate-solubilizing bacteria.”

Discussion :

The long general paragraph on P availability, PSM functions, and soil enzymes belongs to the Introduction, not the Discussion. For exemeple :

- “P is an essential nutrient for plant growth, yet its availability in soil is often limited. Only a small fraction of soil P exists in water-soluble forms that are readily absorbable by crops [44]. The transformation of P within the soil plays a critical role in its uptake and utilization by plants [45]. PSMs which are ubiquitous in agricultural soils, serve as key drivers of the soil P cycle—particularly in the transformation of organic P [10]. These microorganisms facilitate not only the mineralization of organic and microbial P but also the solubilization of insoluble inorganic P, thereby enhancing plant-available P [10]. Soil enzyme activities are widely recognized as early and sensitive indicators of soil responses to perturbations such as microbial inoculation, and reflect broader ecosystem functioning [37,46]. Notably, soil microorganisms can enhance P availability through the production of phytase and related enzymes, which hydrolyze organic P compounds such as phytate [46]. Numerous studies have confirmed that inoculation with PSB or direct application of phosphatases can significantly improve P availability and crop P uptake in low-P soils [47-49]”.

Please move it to the Introduction or rewrite it briefly to focus on interpreting your findings. You may start instead with: “Our results demonstrate that the application of organic P increased…”

- Root characteristic : “Adaptive changes in root morphology play a decisive role in plant P acquisition [51,52]. Increases in root length, surface area, and diameter enhance the capacity of roots to explore soil and absorb P nutrients [53,54]. Due to the low mobility and availability of P in soil, plants largely rely on morphological adaptations—such as increased root length, expanded surface area, and reduced average diameter—to improve P uptake [34,53]. Traits such as adventitious root development, lateral root proliferation, and root hair density further contribute significantly to the acquisition of soil P [55,56].”

The paragraph describing general concepts of root morphology and P acquisition belongs to the Introduction, not the Discussion. Please shorten or move it, and focus on interpreting your own root data.

- Delete “(Fig. 2)” in the Discussion; figures should be referenced mainly in Results.
- Sentences such as “ The PHY treatment led to notable increases in Olsen- P (11.5%) and available potassium (19.9%), indicating its capacity to directly solubilize insoluble P and facilitate potassium release. The PSB treatment significantly elevated organic matter (7.7%), Olsen-P (17.6%), available nitrogen (27.7%), and available potassium (25.9%)” directly repeat Results. Please rewrite to avoid redundancy and interpret results instead of repeating them.
- “….Without sodium phytate, PHY increased organic matter, available P, and available potassium by 18.4%, 85.7%, and 28.0%, respectively, compared to CK” directly repeat Results
- The paragraph beginning with “PSB play a crucial role in P transformation within the rhizosphere, enhancing the bioavailability of otherwise inaccessible P forms and promoting plant nutrient uptake [1]. These microorganisms activate insoluble inorganic P through acidification (e.g., H⁺ release) and carboxylate excretion, while also mineralizing organic P via increased phosphatase and phytase activities [64–66]. For instance, Ramesh et al. demonstrated that PSB inoculation significantly increased soil available P and improved rice growth [57]. Similarly, Chang et al. [20] reported that PSB inoculation increased the labile P pool by 9.2% on average, while moderately labile and non-labile P pools decreased by 6.9% and 5.4%, respectively.” is general background and belongs in the introduction. Please move it or rewrite it briefly to relate it directly to your findings.
